# Genomic analysis of *Xenopus *organizer function

**DOI:** 10.1186/1471-213X-6-27

**Published:** 2006-06-06

**Authors:** Andrew L Hufton, Arunachalam Vinayagam, Sándor Suhai, Julie C Baker

**Affiliations:** 1 Department of Genetics, Stanford University, Stanford, California, USA; 2 Department of Molecular Biophysics, Deutsches Krebsforschungszentrum (DKFZ), Heidelberg, Germany

## Abstract

**Background:**

Studies of the *Xenopus *organizer have laid the foundation for our understanding of the conserved signaling pathways that pattern vertebrate embryos during gastrulation. The two primary activities of the organizer, BMP and Wnt inhibition, can regulate a spectrum of genes that pattern essentially all aspects of the embryo during gastrulation. As our knowledge of organizer signaling grows, it is imperative that we begin knitting together our gene-level knowledge into genome-level signaling models. The goal of this paper was to identify complete lists of genes regulated by different aspects of organizer signaling, thereby providing a deeper understanding of the genomic mechanisms that underlie these complex and fundamental signaling events.

**Results:**

To this end, we ectopically overexpress Noggin and Dkk-1, inhibitors of the BMP and Wnt pathways, respectively, within ventral tissues. After isolating embryonic ventral halves at early and late gastrulation, we analyze the transcriptional response to these molecules within the generated ectopic organizers using oligonucleotide microarrays. An efficient statistical analysis scheme, combined with a new Gene Ontology biological process annotation of the *Xenopus *genome, allows reliable and faithful clustering of molecules based upon their roles during gastrulation. From this data, we identify new organizer-related expression patterns for 19 genes. Moreover, our data sub-divides organizer genes into separate head and trunk organizing groups, which each show distinct responses to Noggin and Dkk-1 activity during gastrulation.

**Conclusion:**

Our data provides a genomic view of the cohorts of genes that respond to Noggin and Dkk-1 activity, allowing us to separate the role of each in organizer function. These patterns demonstrate a model where BMP inhibition plays a largely inductive role during early developmental stages, thereby initiating the suites of genes needed to pattern dorsal tissues. Meanwhile, Wnt inhibition acts later during gastrulation, and is essential for maintenance of organizer gene expression throughout gastrulation, a role which may depend on its ability to block the expression of a host of ventral, posterior, and lateral fate-specifying factors.

## Background

The organizer is the primary patterning center during early vertebrate gastrulation. As might be expected for a tissue with such capabilities, the organizer is complex. Studies in multiple species, including frogs and mice, have shown that the organizer has distinct regions that induce head and trunk, and these abilities decisively change as development proceeds. At the molecular level, the organizer's inductive properties are mediated by factors that inhibit the BMP, Wnt, and Nodal signaling pathways. BMP inhibitors, including the secreted molecule Noggin, can induce a partial secondary axis that lacks a head and notochord. However, BMP inhibition alone cannot sustain the expression of most organizer genes past late gastrula without the addition of Wnt inhibitors, such as Dkk-1 [1]. Furthermore, Wnt inhibitors alone cannot induce secondary structures, but when combined with Noggin can induce a complete secondary axis, including properly patterned head and trunk tissues. Therefore, inhibition of both pathways generates the complete spectrum of molecules required for total organizer function and maintenance, illustrating that regional differences in organizer activity are created by the mixes of inhibitors present and active within particular regions (reviewed in [2-4]).

Research has identified a host of genes that, under the control of the organizer, pattern different aspects of the embryo during gastrulation. As these studies collectively build an ever more complicated tangle of genetic interactions, it is imperative that we begin knitting together our gene-level knowledge into genome-level signaling models. A global analysis can identify comprehensive sets of genes that respond to different aspects of organizer signals i.e. head versus trunk, thus giving us a complete toolbox in which to study the molecular mechanisms regulating organizer function within different contexts and through developmental time. Mapping these genome-level patterns of organizer regulation will allow us to fill-out the current models of gastrula patterning with a greater degree of detail. With these goals in mind, microarray experiments hold particular promise.

Several *Xenopus *microarray-based experiments have been published in recent years as genomic tools have become available. A series of papers have used two-condition comparisons to identify genes up- or down-regulated by a particular process, starting with the cDNA arrays produced by the Brivanlou lab [5,6] and recently using the more comprehensive cDNA arrays developed in the Cho and Ueno labs [7-10]. These methods have been effective in producing new lists of candidate genes, and in two cases have been used to identify genes with new overexpression or morpholino knockdown phenotypes [6,10]. In addition to this two-condition design, studies in other organisms have shown that microarray experiments that employ multiple conditions can be used to cluster genes based on their expression patterns across the samples, and that within these clusters, genes of common function will often group together [11]. This method has been applied fruitfully to the study of specific events in the early development of invertebrates. Some notable examples include the *Drosophila *studies of dorsal-ventral patterning [12] and mesoderm formation [13], where in each case the microarray data was able to subdivide genes based on their roles in these processes. In fact, this type of analysis has recently been applied to the *Xenopus *model; thirty-seven different tissue types were profiled using cDNA arrays creating a broad view of gene expression across development [14]. The resulting cluster data successfully grouped genes with common molecular functions and identified many new tissue specific genes. Moreover, a study by Wessely et al. used an innovative macroarray technique to describe the suites of genes that underlie organizer formation prior to gastrulation, suggesting that genomic methods do have much to offer to early developmental studies [15].

In this paper, we present a genomic view of the signaling processes that underlie certain aspects of organizer function during gastrulation. To this end, we simulated either trunk or head organizer induction by expressing Noggin and/or Dkk-1 within the ventral mesoderm and subsequently analyzed these tissues with oligonucleotide arrays. From this data, we identify genes whose expression levels respond to organizer signaling and then cluster these genes based on their pattern of response. By combining these cluster results with a new GO biological process annotation of the *Xenopus *genome, we are able to rapidly identify clusters that are highly enriched for known gastrula patterning genes. These patterns accurately predict the expression patterns of unknown genes within the clusters: of 19 genes that show a specific pattern during gastrulation, all show organizer enrichment or exclusion in accord with our cluster predictions. Moreover, the cluster patterns allow us to make biological conclusions about the genomic mechanisms that underlie organizer signaling, shedding new light on the divisions between the head and trunk organizer programs.

## Results and discussion

### Creating ectopic organizers with separate functions using Noggin and Dkk-1

**Figure 1:**
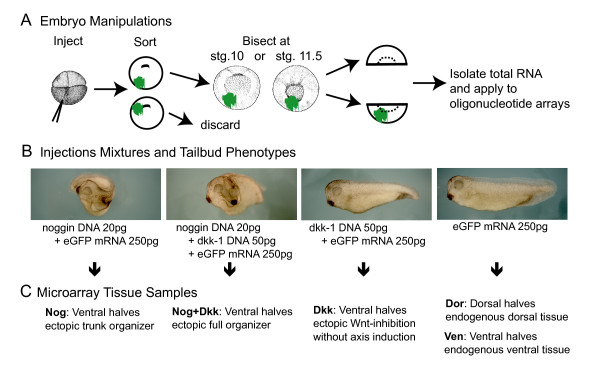
**Generating tissue samples with different aspects of organizer activity**. (A) shows an overview of injection and embryo sorting procedure used to produce samples for microarray analysis. (B) shows the four injection mixtures below their respective tailbud phenotypes. Embryos ventrally injected with these mixtures were bisected at either stg. 10 or stg. 11.5 to produce the tissue conditions in (C).

In order to describe and separate the genomic expression changes induced by the two main organizing activities, BMP inhibition and Wnt inhibition, we ectopically overexpressed one or both of these activities in ventral mesoderm, and compared these samples to endogenous dorsal and ventral mesoderm at early and late gastrula stages. Two well-studied organizer secreted factors, Noggin [16] and Dkk-1 [17], were used to ectopically inhibit BMP and Wnt signaling, respectively. Four different mixtures were injected ventrally into 4-cell embryos: *noggin *and *eGFP *(anti-BMP); *noggin*, *dkk-1*, and *eGFP *(anti-BMP and anti-Wnt); *dkk-1 *and *eGFP *(anti-Wnt); and *eGFP *alone (Figure 1B). *eGFP *mRNA was used to trace targeting. Plasmid DNA was used for both Noggin and Dkk-1 to ensure that these molecules were only expressed after the start of zygotic transcription (mid-blastula transition), thereby mimicking the endogenous regulation of these genes. The ventrally injected embryos were grown to early stage 10, sorted for appropriately targeted eGFP florescence, and then bisected between the dorsal and ventral halves at either stage 10 (early gastrula) or stage 11.5 (late gastrula) (Figure 1A). For the *noggin *and/or *dkk-1 *injected embryos, only the ventral halves of the embryos were saved, eliminating endogenous organizer tissues. For the embryos injected with only *eGFP*, both the ventral and dorsal halves were saved, creating separate ventral (**Ven**) and dorsal (**Dor**) samples. These five conditions (Figure 1C) were each generated twice at both stage 10 (early gastrula) and 11.5 (late gastrula), creating twenty total tissue samples (Table 1). For each batch of injections, remaining sorted embryos were allowed to develop through tailbud stages in order to validate the phenotypes induced by our constructs (Table 1). Total RNA was then isolated from the twenty tissue samples and hybridized to Affymetrix oligonucleotide arrays (see methods).

**Table 1:** Secondary axis phenotypes are improved by sorting for proper targeting

**Clutch**	**Stage**	**Sample**	**Branched NT**	**Normal**
1	10	Nog	17 (100%)	0
1	10	Nog+Dkk	9 (100%)	0
1	10	Dkk	0 (0%)	11
1	10	eGFP Con.	0 (0%)	19

2	10	Nog	16 (100%)	0
2	10	Nog+Dkk	12 (100%)	0
2	10	Dkk	0 (0%)	11
2	10	eGFP Con.	0 (0%)	14

3	11.5	Nog	19 (100%)	0
3	11.5	Nog+Dkk	13 (100%)	0
3	11.5	Dkk	0 (0%)	15
3	11.5	eGFP Con.	0 (0%)	13

4	11.5	Nog	16 (94%)	1
4	11.5	Nog+Dkk	22 (100%)	0
4	11.5	Dkk	0 (0%)	16
4	11.5	eGFP Con.	0 (0%)	15

Sorted embryos assayed at late neurula for the presence of a branched neural tube (NT).

In order to maximize our ability to detect organizer-related expression changes in the microarray data, it was essential that we appropriately control for other sources of biological variability. First, all samples within each replicate were generated from a single clutch of embryos, thereby controlling for genetic heterogeneity in *Xenopus laevis *laboratory populations, which creates observable molecular and morphological differences between clutches. Arima *et al*., using a similar approach, greatly reduced their false detection rate compared to earlier experiments that pooled several clutches in each sample [7]. Second, we reduced variability caused by phenotypic penetrance differences by pre-selecting embryos that showed proper injection targeting. Phenotypic penetrance variation was a concern since both *noggin *and combinations of *noggin *and *dkk-1 *have previously been observed to induce secondary axes in about 50% of ventrally injected embryos [17]. We tested whether this low penetrance could be improved by ensuring accurate targeting to the ventral mesoderm. To this end, we coinjected our constructs with *eGFP *and selected only embryos that contained eGFP fluorescence within the ventral mesoderm at early stage 10. In these sorted embryos, Noggin and Noggin+Dkk-1 were able to induce secondary axes in nearly all the eGFP-sorted embryos (Table 1). Therefore, this sorting protocol (Figure 1A) was used on all the batches generated for microarray analysis and eliminates the concern of phenotypic variability in response to the injected molecules.

These manipulations produce five different tissue conditions, at two gastrula stages, that each contain functionally distinct organizing capabilities (Figure 1 B&C). Ventral mesoderm expressing Noggin (**Nog**) is sufficient to induce secondary trunk tissues. Ventral mesoderm expressing both Noggin and Dkk-1 (**Nog+Dkk**) is sufficient to induce a full secondary axis, including a properly patterned ectopic head. Ventral mesoderm expressing Dkk-1 (**Dkk**) cannot induce any ectopic tissues. Endogenous dorsal tissues (**Dor**) include full organizer capabilities, while endogenous ventral tissue (**Ven**) is used as a baseline control sample throughout the microarray analysis. These samples are designed to contain much informational redundancy in that many gastrula patterning genes should respond to more than one of these conditions. For example, head inducing genes are likely to be up-regulated in both **Nog+Dkk **and **Dor **conditions, when compared to the **Ven **condition. Furthermore, all conditions were collected at both stage 10 and 11.5, bolstering our power to detect organizer-regulated genes, and allowing us to compare early and late responses to organizer signaling.

### Reliable differences in genomic transcription observed in response to Noggin and Dkk-1

**Figure 2:**
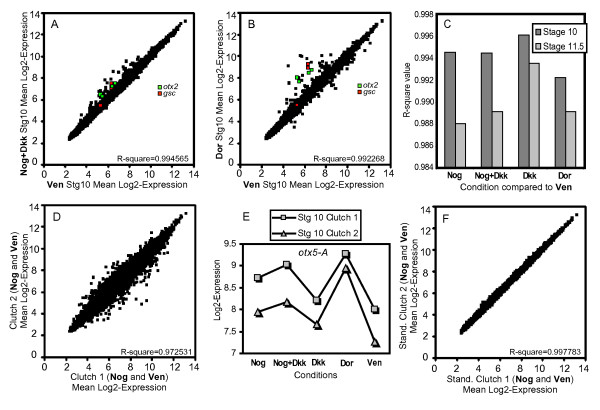
**Expression variation among the samples**. (A) and (B) show scatter plot comparisons of the mean **Nog+Dkk **or **Dor **stg. 10 log_2 _expression values vs the mean stg. 10 **Ven **log_2_expression values. Probe sets measuring two known organizer genes, *otx2 *(green) and *gsc *(red), are labeled within the plots (*otx2 *probe sets: Xl.1268.1.S1_at, Xl.3004.1.A1_at, Xl.11672.1.A1_at, and XlAffx.1.11.S1_at; *gsc *probe sets: Xl.801.1.A1_at, Xl.801.1.S1_at, and Xl.801.1.S1_s_at). (C) R-square regression value summaries for each experimental condition compared to the stage-matched ventral condition. (D) Scatter plot comparison of the mean **Nog **and **Ven **clutch-1 log_2 _expression values vs. the mean **Nog **and **Ven **clutch-2 log_2 _expression values. Note that the clutch variation clearly exceeds differences between the experimental conditions (A-C). Contributing to this variation, many genes show a shift in the average expression between the two clutches, but retain a similar pattern in response to the experimental conditions. As an example, log_2 _expression values for *otx5-A *(Xl.3452.1.A1_at) are shown in (E). After standardizing the average log_2 _expression of both clutches, on a gene-by-gene basis, the comparison from (D) was repeated in (F), eliminating most clutch variation.

To visualize the extent to which each of our experimental treatments induced changes in the population of genes on the array, we used scatter plots and regression analysis. Figure 2A and 2B show comparisons of the averaged stage 10 **Nog+Dkk **or **Dor **samples, versus the averaged stage 10 **Ven **samples (scatter plots for the remaining conditions are shown in Additional file 1 A–F). The majority of the 15,491 probe sets show highly similar expression levels. Nonetheless, there are clearly differentially expressed genes in both comparisons, which in these cases are weighted towards genes that are overexpressed in the **Nog+Dkk **or **Dor **samples. Moreover, there is a greater spread in the **Dor **vs **Ven **plot, which is not surprising since these samples are from distinct endogenous sources. Figure 2C shows a summary of the R-squared values, a regression measure of the correlation between each condition and its stage-matched **Ven **condition. The three conditions that contain axis inducing capabilities (**Nog**, **Nog+Dkk**, **Dor**) show more variation from **Ven **than the **Dkk **conditions, which cannot induce axial tissues. Moreover, the stage 11.5 samples, which have had more time for change to occur, show consistently greater divergence from their stage-matched **Ven **samples than the stage 10 samples. Together these results confirm that organizer signaling differences are driving detectable changes in gene expression within the samples.

To further assess our ability to detect organizer-induced expression changes, we marked probes that correspond to two well-known organizer genes, *otx2 *[18,19] and *goosecoid *(*gsc*) [20], within the previous scatter plots (Figure 2 A&B). Both of these genes are present more than once on the array, thereby also providing a sampling of the consistency among different probe sets. All of the four *otx2 *probes and two of the three *gsc *probes appear to be up-regulated in both the **Nog+Dkk **and the **Dor **samples, as expected. However, in the **Nog+Dkk **plot these inductions appear relatively subtle, highlighting the need for solid statistical methods to select genes that are genuinely altered.

Because of our initial concerns about population heterogeneity in *Xenopus laevis*, we also analyzed the variation between the different clutches used to generate our replicates. To visualize this variation we averaged together the stage 10 **Nog **and **Ven **samples from clutch 1 and compared them by scatter plot to the similarly averaged samples from clutch 2 (Figure 2D), effectively reducing experimentally induced variation, while leaving clutch specific variation (single array comparisons are also shown in Additional file 1 G–I). Clearly, the differences between the clutches exceed the differences between the experimental conditions. However, when we look at the expression patterns of single genes (Figure 2E), we see that both clutches show a similar pattern of expression across the five samples, while the basal level of expression differs between the two clutches. This clutch to clutch shift can be observed in the expression of many genes, however the magnitude and direction of the shift is different for each gene, suggesting that this is not merely an array to array normalization problem. If we subtract out these differences in basal expression, by standardizing the average expression for each gene between the two clutches, we find that these basal shifts explain the majority of the clutch to clutch variation (Figure 2F). Similar patterns were observed in the stage 11.5 data (data not shown). While these clutch differences are striking, organizer signaling induced variation can be easily filtered away from the clutch variation. Replicate averaging removed this source of variation from the scatter plot comparisons between the experimental samples (Figure 2 A&B), and statistical tests can now be conducted by making direct comparisons only between clutch matched samples.

### A successful three-step method that enriches for organizer-related molecules

One of the goals of this study was to functionally subdivide – on a genomic level – genes that are regulated by different aspects of organizer signaling. To this end, we employed a three step computational process to ensure solid statistical analysis and to group genes into clusters that share similar roles during gastrulation.

First, to select genes altered by organizer signaling we used the rank products (RP) method [21]. For each comparison between an experimental sample and its clutch-matched **Ven **control, this method ranks genes by their log-ratio expression difference. Then, the rank numbers from the two replicate sample pairs are multiplied together, producing a 'rank product' score for each gene. From this rank product, a permutation based false detection rate (FDR) can be calculated. This method only makes direct comparisons between clutch-matched samples, eliminating concerns about clutch variation. Moreover, since significance calculations are based on ranks and not absolute values, the method does not assume normality. Up- and down-regulated genes are tested for separately, creating a total of eight statistical tests. Table 2 summarizes the number of genes that passes each test at a 10% FDR. For further analysis, we selected all genes that were significantly altered by overexpression of Noggin and/or Dkk-1, creating a broad list of genes that are likely to be involved in gastrula patterning. At 10% FDR, 188 probe sets are identified that are up- or down-regulated in the **Nog**, **Nog+Dkk**, or **Dkk **conditions, when compared to **Ven**, at either stage 10 or 11.5 (Additional file 2).

**Table 2:** Probe sets declared altered vs Ven by RP test at 10% FDR

Condition	Stage	Increased	Decreased
**Nog**	10	25	0
**Nog+Dkk**	10	47	4
**Dkk**	10	19	0
**Dor**	10	95	54

**Nog**	11.5	62	10
**Nog+Dkk**	11.5	46	34
**Dkk**	11.5	25	47
**Dor**	11.5	109	80

**Figure 3:**
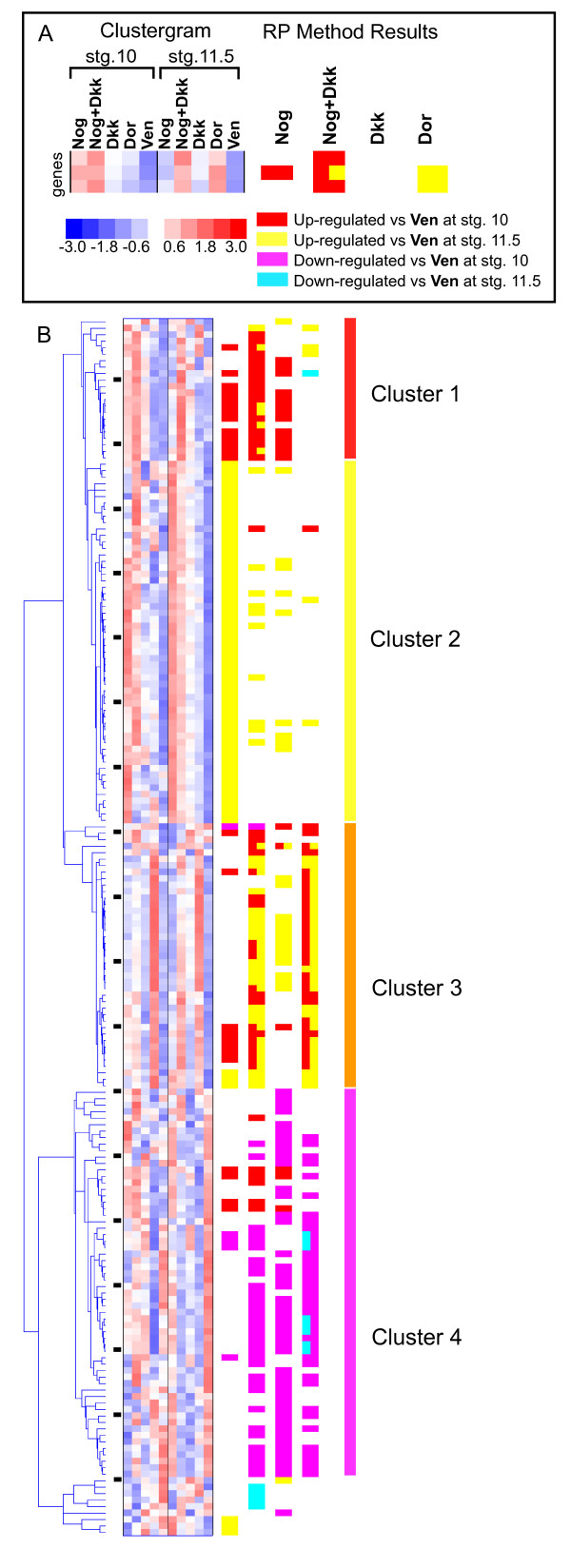
**Clustering genes regulated by organizer signaling**. (A) Key to the hierarchical cluster format used throughout the paper. The clustergram shows the standardized expression intensity for the ten experimental conditions, after replicates have been averaged. To the right of the clustergram, the RP method results at 10% FDR are summarized in four columns representing the comparisons of **Nog, Nog+Dkk**, **Dkk**, or **Dor **to **Ven**. The colors found in the row for each gene represent the tests passed by that gene. Two colors in one column indicate that a gene passed the column's test at both stages. (B) Hierarchical cluster of all the selected genes. The far right shows the hierarchical cluster tree, followed by the clustergram, then RP results. Black ticks between the cluster tree and the clustergram mark every tenth gene, allowing referencing to Additional file 2 for the gene identities. The list break into four main clusters, labeled with red, yellow, orange, and magenta bars. Expanded views of clusters 3 and 4 can be found in Figures 4 and 5, respectively.

Second, hierarchical clustering was performed on this list of 188 probe sets. Replicates were averaged before clustering, and stage 10 and stage 11.5 samples were standardized separately so that the clustering was driven by expression differences between the sample conditions, not the stages. An overview of the entire cluster is shown in Figures 3; Additional file 2 lists the probe set names and their relevant annotations in the same order as the clustergram, allowing referencing to the specific gene identities. The results of the RP method tests at the 10% FDR level are summarized next to the clustergram; a key in Figure 3 explains the colors used to denote these results. By definition each gene has at least one positive test in the first three columns, but many genes passed more than one test. The clustering of these genes divides them into approximately four main groups, with each showing a distinctive pattern of RP test results (Figure 3). The first three groups, containing 119 of the 188 probe sets, are composed of genes that are generally up-regulated compared to **Ven**, while the last group contains the genes that are generally down-regulated compared to **Ven**.

Third, in order to rapidly screen the identified clusters for possible enrichments of developmental processes, we developed a machine annotation method to adopt Biological Process GO annotations from other annotated genomes (see methods). Each of the four main groups from the cluster was then tested for statistically significant enrichments of Biological Process terms (<p = 0.05 after multiple test correction) as compared to the population of genes on the array using the EASE method [22]. The first two clusters show no significant enrichments, while clusters 3 is enriched for 35 terms and cluster 4 is enriched for 27 terms. The top ten terms from each, by p-value, are shown in Table 3. Both clusters share several terms that describe different developmental processes (neurogenesis, morphogenesis, development, organogenesis), and terms related to transcriptional regulation (regulation of transcription from Pol II promoter; regulation of transcription, DNA-dependent; transcription from Pol II promoter). Together these terms indicate strong enrichment for processes involved with development. Moreover, there is a strong correlation in these clusters between the RP test results from the **Nog+Dkk **and the **Dor **conditions, as one would expect from genes that are endogenously relevant to organizer signaling.

**Table 3:** Enriched biological process GO terms

**Enriched GO Biological Process Term**	**p-value**
**Cluster 1**	
No significant enrichments	
**Cluster 2**	
No significant enrichments	
**Cluster 3**	
neurogenesis	9.37E-05
development	1.03E-04
organogenesis	1.48E-04
regulation of transcription from Pol II promoter	1.49E-04
imaginal disc development	1.79E-04
morphogenesis	1.91E-04
transcription from Pol II promoter	2.99E-04
regulation of transcription, DNA-dependent	5.46E-04
regulation of transcription	6.78E-04
regulation of nucleobase, nucleoside, nucleotide and nucleic acid metabolism	7.43E-04
**Cluster 4**	
organogenesis	5.15E-11
morphogenesis	8.04E-11
development	8.59E-11
pattern specification	3.99E-08
neurogenesis	1.24E-07
brain development	2.56E-07
central nervous system development	6.26E-07
regulation of transcription from Pol II promoter	1.96E-06
transcription from Pol II promoter	4.80E-06
regulation of transcription, DNA-dependent	1.04E-05

### Cluster 3 and cluster 4 exclusively contain organizer or ventral-posteriorizing functions, respectively

**Figure 4:**
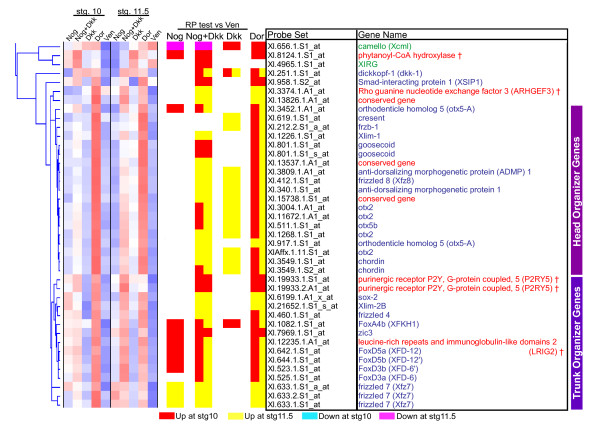
**Cluster 3 is enriched for genes involved in organizer function**. This figure shows an enlarged view of all of the genes in cluster 3, from Figure 3. Each row is annotated with the probe set number and matching gene name. Genes with names in blue have a described role in organizer function. Genes with names in red have no described function during gastrula stage development. Genes with names in green have a published role or expression pattern that is not organizer related. † gene name was assigned by protein sequence homology using the NCBI Homologene database.

**Figure 5:**
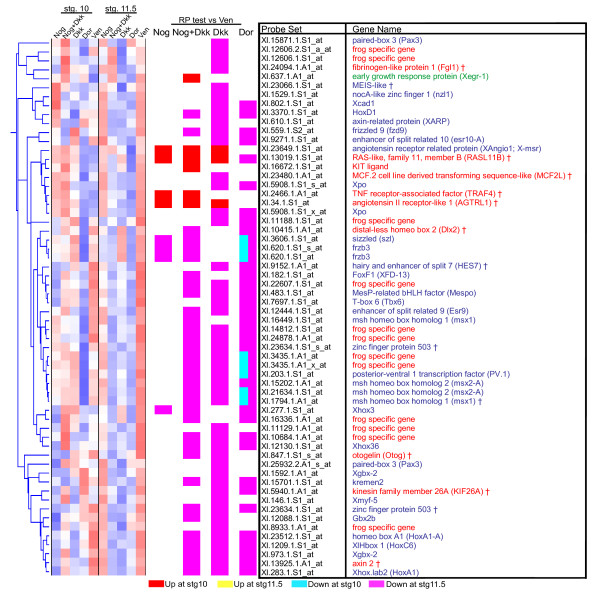
**Cluster 4 is enriched for genes involved in ventral, lateral, and posterior patterning**. This figure shows an enlarged view of all the genes in cluster 4, from Figure 3. Each row is annotated with the probe set number and matching gene name. Genes with names in blue have a described role or specific expression pattern in ventral, lateral, or posterior tissues. Genes with names in red have no described function during gastrula stage development. Genes with names green have a published role or expression pattern that is not ventral, lateral, or posterior related. † gene name was assigned by protein sequence homology using the Homologene database.

In order to both verify our GO annotation descriptions and to provide a deeper understanding of the biological processes behind these clusters, we searched the literature for publications relating any of these genes to early development. Fitting the GO predictions, among clusters 1 and 2 we found only a handful of known developmental regulators:*fibroblast growth factor receptor 2 *[38], *secreted frizzled-related sequence protein 2 *(*sfrp2*) [39], and *mix.3 *(*mixer*) [13] (Additional file 2). Furthermore, clusters 3 and 4, which the GO annotations identified as significantly enriched for developmental terms, contain many published developmental regulators. Cluster 3 is densely populated with genes that have known roles in organizer function (Figure 4). Of the 31 unique genes in this cluster, 22 (71%) have described roles in organizer function. Of the 9 remaining genes, only 2 have described roles in early development that are not clearly organizer related, and both of these genes are distinct outliers to the cluster group. Moreover, cluster 4 is largely comprised of genes involved in gastrula and neurula stage patterning of ventral, lateral, and posterior tissues – all tissue types that are repressed by organizer activity (Figure 5). Of the 52 unique genes in this cluster, 31 (60%) have a described role in the patterning of these tissues. 27 are known or predicted transcriptional regulators, of which 17 encode homeobox proteins, highlighting the importance of these genes in developmental patterning. This cluster also contains a small sub-cluster of genes with an interesting and unexpected pattern. These genes are induced at stage 10 in the three ectopic organizer conditions,**Nog**, **Nog+Dkk**, and **Dkk**, but are strongly down-regulated in the stage 11.5 **Dor **condition. Within this sub-cluster is the G-protein coupled receptor gene *X-msr (Xangio1)*, a known marker of paraxial mesoderm [23], as well as two unstudied genes encoding a paralogous G-protein coupled receptor (Xl.34.1.S1_at), and a ras-like protein (Xl.13019.1.S1_at), suggesting that perhaps this cluster contains components of an unknown signaling process. Overall, a combination of GO annotations and literature validation has revealed two clusters of genes within our dataset that represent specific and discrete functions occurring during gastrula: organizer function and dorsal patterning (cluster 3); and ventral, lateral, and posterior patterning (cluster 4).

### Cluster 3 and cluster 4 reveal 12 new genes with organizer-related expression patterns

**Figure 6:**
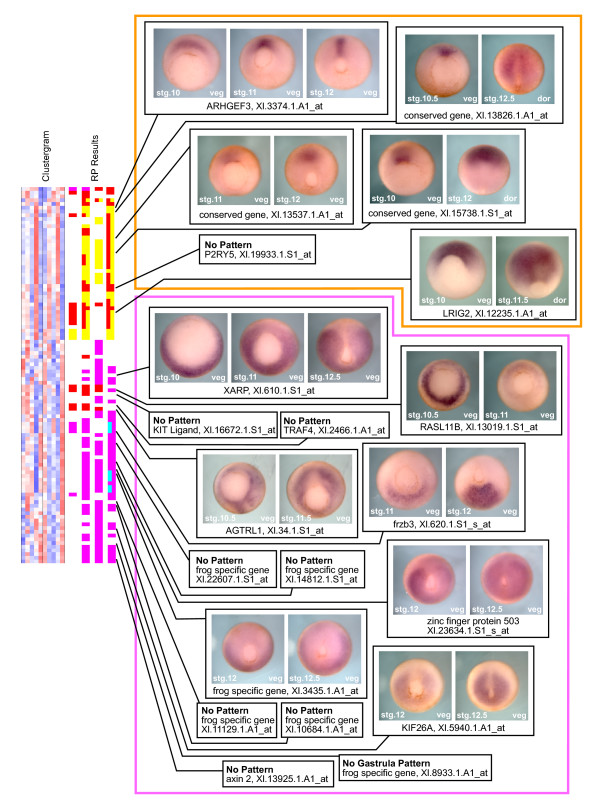
**Clusters 3 and 4 faithfully predict expression patterns for unknown genes**. Genes found in clusters 3 and 4 that lacked described gastrula stage expression patterns were analyzed by whole mount *in situ *hybridization. Unknown genes from cluster 3 that showed a specific pattern are enriched in organizer tissues (orange box), and unknown genes from cluster 4 that showed a specific pattern are excluded from organizer tissues (magenta box). Each tested gene is labeled with its name and the Affymetrix probe set number. Genes marked "no pattern" showed no staining, or a non-specific staining pattern that was similar to sense controls. Genes marked "no gastrula pattern," showed no pattern during gastrula stages, but did show specific patterns at later stages that are not shown here. Each photo is labeled with the developmental stage of the embryo in the bottom left corner, and the orientation in the bottom right corner. veg: vegetal view, dorsal faces up. dor: dorsal view, anterior faces up.

Since this method of clustering clearly enriches for known organizer-related genes, our next step was to test whether our clustering method could also successfully identify unknown genes involved in these processes. To that end, we selected genes without known gastrula-stage expression patterns from our two developmentally enriched clusters and analyzed each gene by whole mount *in situ *hybridization. We obtained clones for twenty-one genes: six clones from the organizer gene rich cluster 3, and fifteen clones from the ventral/posterior/lateral gene rich cluster 4. *In situ *results are shown in Figure 6. Of the 12 clones that showed clear patterns during gastrulation, all clones from cluster 3 showed organizer enrichment, and all clones from cluster 4 showed organizer exclusion. Moreover, there appear to be finer correlations between some of the *in situ *patterns and the location of the genes within the cluster. For example, both *Xl.13537.1.A1_at *and *Xl.15738.1.S1_at *show expression that starts in the dorsal lip and then migrates anteriorly with the developing head tissues, displaying patterns that are similar to their immediate flanking neighbors in the cluster, *gsc *[20] and *otx2 *[18,19]. Overall, these clusters faithfully classify transcripts by their gastrulation expression.

The majority of the unknown genes within these clusters have no previous associations with development and no known function. However, several have previous links that implicate them in developmental patterning, and our expression patterns are consistent with these previous findings. A clone related to *LRIG2 *was recently identified in a microarray screen for neural specific genes, and showed an identical expression pattern to our clone (NIBB clone XL098p21 [9]). *AGTRL1 *and *frzb3 *each have close paralogs in *Xenopus *that have described expression patterns identical to our genes: *X-msr *[23] and *sizzled *[24] respectively. Both of these described genes pass the RP method test and cluster near their undescribed paralog (Figure 5). Additionally, two genes for which we described new ventral-posterior expression patterns, *zinc finger protein 503 *and *XARP*, produce proteins that have previous associations with posterior patterning. Zinc finger protein 503 belongs to a family of NocA-like zinc-finger proteins that have been implicated in zebrafish hindbrain patterning [25-27], and XARP is the *Xenopus *ortholog of mammalian axin 2, which plays an important role in the transduction of the canonical Wnt signaling cascade [28], a key posteriorizing signal during gastrulation.

### Relaxing stringency on genes similarly expressed within Nog+Dkk and Dor conditions reveals seven new organizer-related molecules

In our initial clustering results, most known gastrula patterning genes were similarly induced or repressed in the two conditions with complete organizer function, **Nog+Dkk **and **Dor**. Furthermore, all new expression patterns identified from clusters 3 and 4 were consistent with our expectations, indicating that the clusters were highly reliable (Figure 6). Based on these observations, we hypothesized that among genes with similar expression in the **Nog+Dkk **and **Dor **conditions we should be able to relax our statistical test and identify additional unknown organizer-related genes. To that end, we selected for genes with a RP score of less than 0.0006 in both the **Nog+Dkk **and the **Dor **conditions, when compared to **Ven**, at either stage 10 or stage 11.5. The test was repeated for both up-regulated and down-regulated genes and then all positive genes were merged, creating a new list of 220 probe sets (Additional file 3). Because the two tests that we require each gene to pass are not independent, there is no clear way to calculate a FDR for this list of genes.

**Figure 7:**
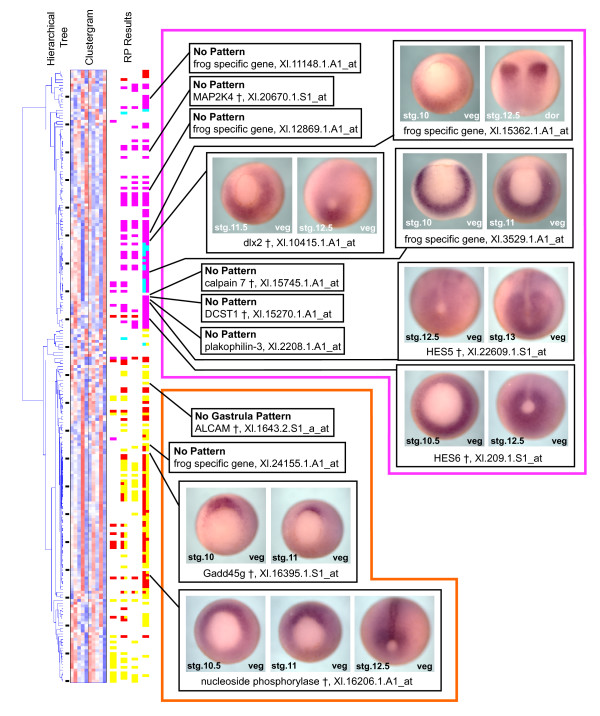
**Relaxed statistical criteria select for additional genes with organizer-related expression patterns**. The cluster results for the second set of RP criteria, which required correlated expression in the **Nog+Dkk **and **Dor **conditions, identifies additional unknown genes with organizer-related expression patterns. The hierarchical tree is on the far left, followed by the clustergram, and then the summary of the RP results at 10% FDR. Colors and columns are same as described in the Figure 3A. Black ticks between the cluster tree and the clustergram mark every tenth gene, allowing referencing to Additional file 3 for the gene identities. Genes in this list that lacked described gastrula stage expression patterns were analyzed by whole mount *in situ *hybridization. The top of the cluster contains genes that were repressed in the **Nog+Dkk **and **Dor **conditions; unknown genes within this group are excluded from organizer tissues (magenta box). The bottom of the cluster contains genes that were activated in the **Nog+Dkk **and **Dor **conditions; unknown genes within this group are enriched in organizer tissues (orange box). Each tested gene is labeled with its name and the Affymetrix probe set number. Genes marked "no pattern" showed no staining, or a non-specific staining pattern that was similar to sense controls. Genes marked "no gastrula pattern," showed no pattern during gastrula stages, but did show specific patterns at later stages that are not shown here. Each photo is labeled with the developmental stage of the embryo in the bottom left corner, and the orientation in the bottom right corner. veg: vegetal view, dorsal faces up. dor: dorsal view, anterior faces up. † gene name was assigned by protein sequence similarity using Homologene.

Since this second list of genes was specifically required to be similarly up- or down-regulated in the **Nog+Dkk-1 **and **Dor **conditions, hierarchical clustering of these genes produces a clustergram with less pattern variety (overview in Figure 7 and probe information in Additional file 3). Broadly, this cluster retains most of the genes from the first list's clusters 3 and 4, while eliminating most of the genes from clusters 1 and 2 (Figure 3). Additionally, this new list captures 119 new probe sets not represented in the first. Many of these are known gastrula patterning genes, including important organizer regulators such as *Xnot *[29] and *cerberus *[30], and ventral fate inducers that are repressed by organizer signaling, such as *vent-2 *[31], *vox-1 *(*Xbr-1*) [32], *Xvex-1 *[33]. Hence, our second set of RP criteria does indeed find more organizer-regulated genes, and it also eliminates the clusters of genes in the first list which have no clear relevance to gastrula patterning.

From this list of genes we selected fifteen additional clones to test by *in situ *hybridization, again predicting either organizer-enriched or organizer-excluded expression (Figure 7). Of the seven genes that showed clear patterns during gastrula stages, once again, the microarray data correctly predicts organizer enrichment or organizer-exclusion for all. Of these seven genes, four have no known developmental function, and the remaining three, HES5, HES6, and dlx2, have described functions during neurulation [34-36].

### Noggin and Dkk-1 regulate newly identified genes in patterns that validate the microarray data

**Figure 8:**
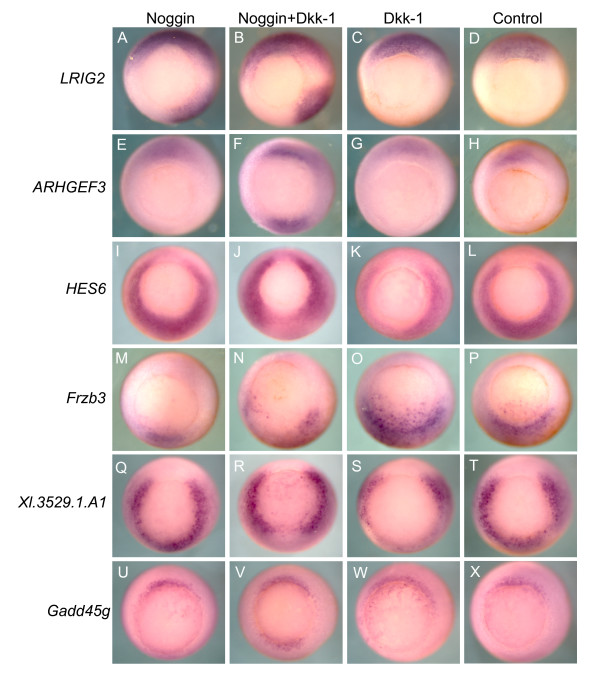
**Noggin and Dkk-1 regulate the expression of newly identified genes**. 4-cell embryos were ventrally injected with the *noggin *and/or *dkk-1 *concentrations described in Figure 1, and tested by *in situ *hybridization for patterns of ectopic induction or repression. In each case the observed *in situ *patterns confirm the microarray patterns (A-D) *LRIG2 *shows ectopic ventral expression in both the Noggin and the Noggin+Dkk-1 overexpressing embryos, but not in the Dkk-1 embryos. (E-H) *ARHGEF3 *shows ectopic ventral expression only in the Noggin+Dkk-1 overexpressing embryos. (I-L) *HES6 *shows ectopic ventral repression in the Noggin+Dkk-1 and the Dkk-1 overexpressing embryos. (M-P) *Frzb3 *expression is ectopically repressed by Noggin or Noggin+Dkk-1 overexpression. (Q-T) *Xl.3529.1.A1 *expression is ectopically repressed by Noggin+Dkk-1 and Dkk-1 overexpression. (U-X) *Gadd45g *is ectopically induced only in the Noggin+Dkk-1 condition. All embryos are between stages 10.5 and 11 and are shown in vegetal view with dorsal side facing up.

To further assure that we were genuinely identifying genes regulated by organizer signaling, we selected several of the unknown genes identified by the clustering results and analyzed their expression patterns in stage 10.5 embryos overexpressing Noggin and/or Dkk-1. *LRIG2 *(Figure 8 A–D), and *ARHGEF3 *(Figure 8 E–H) both showed strong ectopic ventral expression in embryos overexpressing Noggin+Dkk-1, but only *LRIG2 *showed ectopic expression with Noggin alone, matching the microarray data. *HES6 *expression (Figure 8 I–L) was successfully cleared from ectopic ventral regions by Noggin+Dkk-1 and Dkk-1, but not Noggin alone, again recapitulating the microarray data. *Frzb3 *expression was disrupted by Noggin and Noggin+Dkk-1 overexpression, as the microarray data reported (Figure 8 M, N). Moreover, in some cases *Frzb3 *appeared slightly expanded by Dkk-1; the microarray recorded a weak induction by Dkk-1 at stage 10, but not stage 11.5 (Figure 8 O). The expression patterns of *Xl.13826.1.A1_at *and *RASL11B *were not clearly altered by ectopic Noggin and/or Dkk-1 (data not shown). However, *Xl.13826.1.A1_at *produces an extremely faint stain, and *RASL11B *exhibits a fair amount of spottiness and variability among wild type embryos, making it difficult to convincingly identify ectopic pattern alterations in either.

From the genes identified by the second clustering effort, *Xl.3529.1.A1 *showed disruption by Noggin+Dkk-1 and Dkk-1, but not Noggin, as expected (Figure 8 Q–T), and *gadd45g *only shows induction by the combination of Noggin+Dkk-1 (Figure 8 U–X). On the array, *gadd45g *has no inductions that break 10% FDR cutoff, but it was selected by our second set of RP criteria because of correlated inductions in both the **Nog+Dkk **and **Dor **conditions. This again confirms that the more focused set of RP criteria is successfully identifying genuine organizer-regulated genes that fell below the more stringent 10% FDR cutoff.

### Head and trunk organizing genes show distinct responses to Noggin and Dkk-1

In addition to allowing us to identify new organizer-regulated genes, the cluster patterns provide us with a broad overview of the genomic response to organizer signaling, allowing us to compare these patterns with current models of organizer function. A close inspection of cluster 3 shows that the genes seem to subdivide in two groups that represent the head and trunk organizers (Figure 4). The top two-thirds of the cluster is dense with genes that can induce head identity (*otx2 *[18,19], *otx5 *[37], *frzb-1 *[38]) and includes some genes that are sufficient to induce secondary axes (*gsc *[20], *chordin *[39]). The bottom of the cluster contains genes that are strongly induced by Noggin alone, and indeed many of these genes produce proteins that are implicated in processes crucial to trunk formation: three Fox proteins, A4, D3, and D5, which pattern or induce different aspects of trunk tissues (reviewed in [40]); Zic3, a potent neural inducer [41]; and Frizzled 7, a key player in convergent extension [42]. A key difference between these sub-clusters is their differing response to Noggin in the absence of Dkk-1. The head sub-cluster shows some weak induction by Noggin at stage 10, but this induction never crosses the 10% FDR cutoff, and no measurable induction remains for these genes by late gastrulation (Figure 4). In contrast, the trunk sub-cluster shows strong early induction by Noggin, and most genes show some induction in late gastrula embryos, although all are below the 10% FDR cutoff.

**Figure 9:**
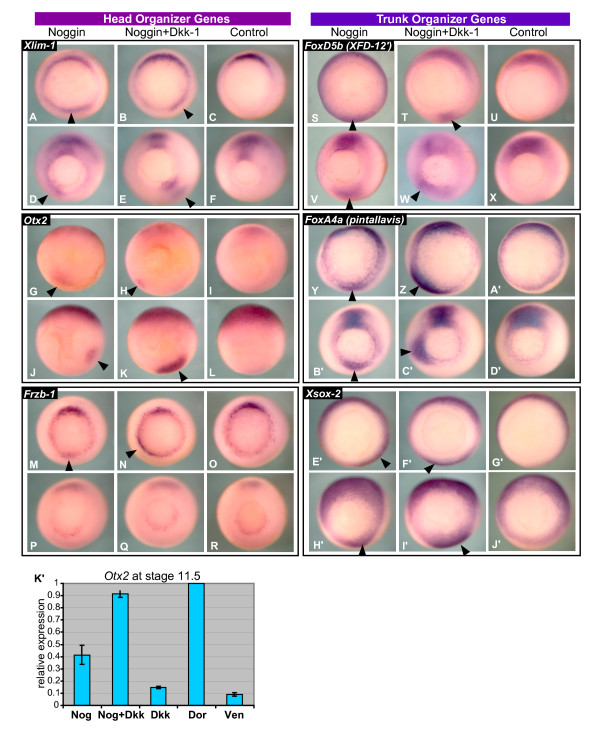
**The head and trunk sub-cluster genes show distinct responses to organizer signaling**. Three genes were selected from each sub-cluster, and tested by *in situ *hybridization at stage 10.5 (top row) and 11.5 (bottom row), in embryos ventrally injected with *noggin *or *noggin+dkk-1*. Black arrowheads mark ectopic staining. *Xlim-1 *(A-F) and *otx2 *(M-R) expression are similarly induced at stage 10.5 by Noggin and Noggin+Dkk-1, but by stage 11.5 Noggin+Dkk-1 induction is clearly much stronger and more widespread. For *Xlim-1*, ectopic expression induced by Noggin+Dkk-1 is observed migrating away from the blastopore lip region, but never for Noggin alone. (M-R) *Frzb-1 *expression is ectopically induced only by the combination of Noggin+Dkk-1, not Noggin alone, and neither can sustain expression into late gastrulation. For the three trunk genes, *FoxD5b *(*XFD-12'*) (S-X), *FoxA4a *(*pintallavis*) (Y-D'), and *Xsox-2 *(E'-J') ectopic induction is similar in both intensity and spread in Noggin and Noggin+Dkk-1 overexpressing embryos at stage 10.5 and 11.5. A-L and S-J' vegetal view; M-R animal view. Dorsal faces up in all pictures. (K') Otx2 expression was assayed by real-time RT-PCR, in stage 11.5 ventral tissues injected with the same mixtures used to create the microarray samples. Weak, but significant (p = 0.043 by one-sided *t*-test), induction in Dkk-1 overexpressing tissues are seen compared to ventral, supporting the weak Dkk-1 inductions seen on the microarray. Error bars show the standard error calculated from two biological replicates.

To confirm these cluster sub-divisions and better visualize how the genes from the head and trunk sub-clusters differed in their response to organizer signaling, we performed *in situ *hybridization on genes from each cluster in *noggin *and *noggin+dkk-1 *injected embryos during both early and late gastrulation (Figure 9 A–J'). Two genes from the head sub-cluster, *Xlim-1 *and *otx2*, show similar weak ectopic induction by both Noggin and Noggin+Dkk-1 at stage 10.5, but by stage 11.5 the induction is much stronger and more widespread in the Noggin+Dkk-1 overexpressing embryos (Figure 9 A–L). Indeed, for *Xlim-1*, ectopic expression induced by Noggin alone never expands past the immediate border of the blastopore lip region (Figure 9 A–F). For these head organizer genes Dkk-1 seems to play a critical role in maintaining induction throughout gastrulation. We also tested *frzb-1*, a member of the head sub-cluster, which, in contrast to most of the head cluster genes, is not significantly induced by Noggin or Noggin+Dkk-1 on the array, although weak inductions are recorded in each condition. In accord with these measurements, we can see some weak ectopic staining during early gastrulation, however these induction disappears completely by stage 11.5 (Figure 9 M–R).

From the trunk genes, we tested two trunk patterning genes *foxD5b *(*XFD-12'*) and *foxA4a *(*pintallavis*), as well the general neural inducer *Xsox-2*. *FoxA4a *is a close paralog, and likely pseudoallele, of *FoxA4b *(*XFKH1*) which is among the trunk sub-cluster genes (Figure 4). Both genes show the same developmental expression pattern (reviewed in [40]). *FoxA4a *is captured by our second set of RP criteria, and groups among the trunk cluster genes found by the first gene list (Additional file 3). For these three trunk genes, the ectopic expression intensity and territory is more similar between the Noggin and Noggin+Dkk-1 overexpressing embryos, than for the head cluster genes (Figure 9 S–J'). Together these results indicate that our clustering results are genuinely subdividing genes into groups with different responses to organizer signaling, and these patterns give us a better understanding of how organizer function is generated at the genomic level.

Another key difference between the head and trunk sub-cluster genes are the weak Dkk-1 inductions observed for several head organizer genes. Conflicting with the array data, Dkk-1 injected embryos did not show ectopic staining for *frzb-1 *and *otx2 *by *in situ *hybridization (data not shown). To test whether these inductions might simply be below the level of detection in our *in situ *analysis, we used real-time RT-PCR to analyze *otx2 *expression in response to ectopic Noggin and/or Dkk-1. Embryos were injected, sorted, and dissected by the same protocol used to generate the array samples, creating two new sets of tissue samples at stage 11.5. Real-time RT-PCR analysis shows that Dkk-1 does indeed induce *otx2 *in these samples, with an average induction of 1.65-fold over ventral (Figure 9K'; p = 0.043 by one-sided *t*-test). This is in line with the inductions seen on the array; the four *otx2 *microarray probe sets show an average induction of 1.99-fold over ventral. This supports the reliability of the array data, and shows that Dkk-1 does have some ability to weakly induce organizer genes in the ventral tissues of whole embryos without ectopic anti-BMP signaling.

## Conclusion

In these studies we used our existing knowledge of organizer function as leverage in the design and analysis of a genomic visualization of gastrula stage signaling. To this end, we ectopically expressed Noggin and Dkk-1 to induce different aspects of organizer activity, and then analyzed the genomic expression consequences of these activities at two different gastrula stages. The resulting data provide us with two valuable sources of information. First, our analysis generates clusters that are strongly enriched for known gastrula patterning genes. Using these enrichments we were able to predict with high confidence the expression patterns of unknown genes within the clusters. Second, our results provide a genome-level view of the transcriptional response patterns to organizer signaling, which helps us to understand the separate roles played by BMP and Wnt inhibition during organizer function, and also defines suites of genes that share similar response patterns.

### A genomic view of organizer signaling refines our understanding of organizer function

Overall our data reveals that most gastrula-patterning genes respond to organizer signaling according to a few distinct patterns. These patterns divide genes into approximately four groups: a) Trunk patterning genes that are primarily induced by Noggin; b) Head and general organizer genes that require both Noggin and Dkk-1 for strong induction, and in some cases show weak induction by Dkk-1 alone; c) A small number of genes that are primarily repressed by Noggin; d) A wide range of ventral and posterior genes that are primarily repressed by Dkk-1. These broad patterns help to elucidate the genomic networks that underlie organizer signaling and provide a deeper functional understanding of different phenotypes induced by BMP and Wnt inhibition.

The group of genes primarily induced by Noggin appears to be sufficient to explain the secondary trunk tissues induced by BMP inhibition, a phenomenon that has remained somewhat mysterious in light of previous reports that did not observe sustained induction of organizer genes by BMP inhibition [1]. This cluster contains a set of Fox genes that can induce and pattern various aspects of trunk identity [40], as well as a strong neural inducer, Zic3 [41], and Frizzled 7, a part of the non-canonical Wnt signaling pathway that regulates convergent extension movements [42]. Noggin strongly induces these genes at stage 10, and maintains induction of these genes into late gastrulation. This late induction is seen weakly on the microarray, but presents clear ectopic staining by *in situ *hybridization (Figure 9 S–J'). Indeed, in concert with previous reports, we observe that Noggin induction of head and general organizer molecules is comparatively weak, becoming undetectable on the microarray by stage 11.5, clearly explaining Noggin's inability to induce head structures on its own. In contrast, Wnt inhibition by Dkk-1 can weakly induce several organizer genes at 11.5, including secreted BMP and Wnt inhibitors like *chordin *and *frzb-1*, but has no similar inductive capabilities on the trunk cluster genes we identified. Hence, we have isolated a group of trunk-inducing genes that share a distinctly regulatory pattern that is less sensitive to Wnt signaling than other organizer factors. These genes may be the key to understanding Noggin's ability to induce secondary tissues in the absence of Wnt inhibition.

In addition to helping us understand BMP inhibition's induction of trunk tissues, the patterns seen here reveal a clear difference in the role Wnt and BMP inhibition play in repressing gene expression. Overexpression of Dkk-1 represses a wide-range of ventral, posterior, and lateral genes, more in fact than the combination treatment of Noggin+Dkk-1. Although the essential role Wnt signaling plays in posteriorizing embryos is well appreciated [43], the sheer number and variety of genes repressed by Dkk-1 is quite surprising, especially in contrast to Noggin, which can only significantly repress three genes, *sizzled*, *frzb3*, and *Xhox3*. Interestingly, none of these three genes can be repressed by Dkk-1, highlighting the functional separation of BMP and Wnt inhibition. In fact, Dkk-1 is even a better repressor of well-described BMP targets like *msx1 *(Nog -1.06 fold; Dkk -1.58 fold at stage 11.5) [44]. Together these results indicate that during gastrulation Wnt inhibition is largely responsible for blocking the spread of activities from outside of the dorso-anterior domain, thereby preventing the ventralization and posteriorization of the organizer. Moreover, for many genes, this activity appears to be essentially independent of BMP inhibition. Taken together, these results help to explain the critical role anti-Wnts play in maintaining organizer gene expression as gastrulation progresses.

Although individually Noggin and Dkk-1 display distinct and separate activities during gastrulation, the combination of Noggin and Dkk-1 generates tissue expressing both suites of organizer genes: the trunk patterning genes induced early by Noggin, and the head organizer genes weakly induced late by Dkk-1. Furthermore this combinatorial induction is more than additive; many of these genes are more strongly induced during both early and late gastrulation, overcoming the temporal restrictions that are evident with either activity alone. This duality of signaling via BMP and Wnt inhibitory actions has been previously indicated using single gene approaches: the competence of ectopic BMP inhibition to induce secondary axes is known to end abruptly at stage 10 [45,46], and it can only maintain organizer gene inductions in the presence of anti-Wnt signals [1]. Our data extends these observations to a genomic level, describing suites of genes that underlie these observed phenotypes. These patterns clearly demonstrate distinct temporal roles for Noggin and Dkk-1 in the establishment of the complete organizer.

Overall, analysis of the differences in the expression changes induced by Noggin and Dkk-1 supports a model where BMP inhibition plays a largely inductive role during early developmental stages, thereby initiating the suites of genes needed to pattern dorsal tissues. Meanwhile, Wnt inhibition acts later during gastrulation, and is essential for maintenance of organizer gene expression throughout gastrulation, a role which may depend on its ability to block the expression of a host of ventral, posterior, and lateral fate specifying transcription factors. By observing these genomic mechanisms behind known developmental phenomenon we are helping to move toward a network understanding of organizer function.

### Statistical approach vastly enriches for genes expressed in organizer related patterns during gastrulation

In order to detect genes that were regulated by organizer signaling, we employed the rank products (RP) method [21], which we believe represents a significant advance over previous statistical testing methods. Common *t*-test based methods test each gene on the array separately, and then must make a multiple test correction for the tens of thousands of genes tested, greatly reducing their ability to detect significant change. In contrast, the RP method uses a simple ranking strategy that looks at all genes relative to one another. In essence, this is a much more realistic model of genomic regulation, since the expression levels of all genes are interconnected. Hence, we benefit from the sheer number of genes on the array; genes that rank themselves near the top of a list with tens of thousands of members in more than one replicate are highly likely to be significantly altered. By using the RP method for our statistical tests we were able to produce acceptable FDR rates with only two replicates, freeing us to complete more conditions.

Next, we classified the identified genes based on their pattern of response to the different conditions. Our experiment contains a panel of conditions that represent distinct aspects of organizer signaling, helping to focus our hierarchical clustering results on biologically relevant gastrula stage processes. We found that genes known to be involved in organizer function clustered tightly together (Figure 3, cluster 3) and genes that induce ventral, posterior, and lateral fates clustered separately (Figure 3, cluster 4). Moreover, genes that have no clear relationships to development were largely segregated from the known gastrula patterning genes (clusters 1 and 2, Figure 3), highlighting the value of combining a statistical test with hierarchical clustering.

Overall, this strategy was highly successful at enriching for known organizer genes and predicting the expression patterns of unknown genes. From the first broad set of RP criteria (Figure 3), 70% of the cluster 3 genes already have described organizer-related functions, and 60% of the cluster 4 genes are known to play a role in the patterning of lateral, posterior, or ventral fates, functions that oppose the dorsalizing and anteriorizing influences of the organizer (Figure 4 &5). Using these enrichments of known gastrula patterning genes, we were able to predict the expression patterns of unknown genes within the clusters with remarkable accuracy. In all cases, genes that showed a specific *in situ *pattern during gastrula stages were either organizer-expressed or organizer-excluded in a manner consistent with their cluster. Once we account for these new patterns, we find that 87% of the genes in cluster 3 are functionally important to, or at least have increased expression in, the organizer. Similarly, 73% of the genes in cluster 4 have functions that oppose organizer activity, or expression that is organizer-excluded. These numbers are conservative since we did not test every unknown gene within these clusters, and some of our negative *in situ *results probably resulted from clones that produced poor probes (*in situ *false negatives), rather than from microarray measurement errors (microarray false positives). Moreover, the total lack of *in situ *patterns contrary to our expectations suggests that our clustering predictions were highly reliable. Overall, these studies reveal new restricted gastrula expression patterns for 19 genes, 11 of which lacked any previous associations with early development, providing a list of candidates for future functional studies.

While our data did provide a powerful enrichment for gastrula patterning genes, it is clear the some key genes were missed in our analysis. For instance BMP-4, the ventral signal that is inhibited by Noggin and other BMP inhibitors, did show lower expression in the **Dor **samples, but the difference was not declared significant by our statistical test. Additionally, several key ventralizing molecules that have previously been shown to be repressed by BMP inhibition, *vent-2*, *vox-1 *(*Xbr-1*), and *Xvex-1*, were not captured by our main clustering list, although they were captured by our second less stringent gene list (Additional file 3) [31-33]. Indeed, for these genes we do observe weak repression by Noggin activity alone (mean = -1.26 fold at stage 11.5). Together, these data indicate that there are real expression differences relevant to organizer signaling that fall below our statistical cutoffs. Additionally, our data identifies many genes that were significantly different between the **Dor **and **Ven **samples, but were not affected by Noggin or Dkk-1 overexpression, and as such were not analyzed in this paper. These include several genes important to early developmental patterning such as *X*w*nt-8*, *Xnr3*, and *siamois *[3,4].

### Adoption of GO annotation from other organisms allows rapid identification of developmental gene enrichments

In this paper we have used machine assigned annotations to help identify clusters enriched for development functions, and to give objective measures of significance to these enrichments. This has helped us to address a cardinal challenge in the analysis of microarray data: the need to sift through long lists of genes and glean common functional themes, a laborious and subjective process. This challenge has been a primary motivator in the development of systematic gene annotation schemes such as the Gene Ontology (GO) [47]. The *Xenopus *genome has not been directly annotated using these systems, but methods have been published that allow the adoption of gene annotations from related genes in other organisms, including the method previously described in Vinayagam *et al*. [48]. For this paper we used a modification of this method to generate biological process GO annotations for the *Xenopus *genome. Not surprisingly, the metrics produced in building this annotation suggest that biological process terms are more difficult than molecular function terms to map between organisms based on protein sequence similarity. Regardless, after employing a strict statistical cutoff to select only terms assigned with high confidence, we found that this annotation provided a useful method to rapidly identify clusters that were enriched for developmental processes, and the p-values proved that we were receiving highly significant enrichments.

### Future directions

Our results provide new candidate genes for functional studies, and describe the transcriptional response to organizer signaling on a genomic scale. Currently, we are testing the new organizer-regulated genes for specific functions during gastrulation using traditional overexpression techniques. More broadly, we hypothesize that the suites of commonly regulated genes described by our clusters may share similar regulatory mechanisms. To explore this possibility we are investigating methods that combine our microarray patterns with regulatory element prediction algorithms, using the *Xenopus tropicalis *genome to provide flanking sequence for each gene. As these resources grow we hope to begin building a rigorous, network-based model of gastrula patterning. Lastly, we have presented and discussed only a small portion of the data generated by our experiment. The raw data from our arrays has been made available through the NCBI Gene Expression Omnibus (GEO) database [49] (GSE3368), with the hope of providing utility for researchers beyond the conclusions in this paper.

## Methods

### Embryology and overexpression

Female adult *Xenopus *laevis were ovulated by injection of human chorionic gonadotropin, and eggs were fertilized *in vitro *[50]. After treatment with 2.5% cysteine (pH 8.0) embryos were reared in 1/3 MR. For microinjections, embryos were placed in 2.5% Ficoll in 1/3 MR and injected at 4-cell stage into the marginal zone of one ventral blastomere. Each embryo received 5 nl of a solution containing a combination of *noggin *(pCS2noggin) and/or *dkk-1 *(pCSdkk-1 [17]) plasmid and *eGFP *mRNA (concentrations in Figure 1), in sterile RNase-free water. Plasmid constructs were linearized at NotI sites and purified by incubation with 0.1μg/μl proteinase K and 0.5% SDS, followed by phenol:chloroform:isoamyl alcohol extraction and sodium acetate/ethanol precipitation. *eGFP *mRNA was prepared using the Sp6 mMessage mMACHINE kit (Ambion) from the CS2P eGFP X/P plasmid. Embryos produced for microarray analysis were sorted at early stage 10 for eGFP fluorescence that was located ventral-marginal, using the emerging blastopore lip to differentiate dorsal and ventral hemispheres. For stage 10 samples, embryos were bisected when the blastopore lip had spread about 50% around the embryo, again using the blastopore lip to distinguish the dorsal and ventral halves. For stage 11.5 embryos there is no reliable morphological indicator of dorsal-ventral polarity, so embryos were cut using eGFP fluorescence as a marker of ventral identity. Embryos were manually devitellinized, followed by bisection with a scalpel blade. Bisected embryo halves were dropped into a microfuge tube resting in liquid nitrogen within a minute of cutting. Ten embryo halves were collected for each sample condition, and stored at -80°C until RNA extraction. Each batch of five conditions was conducted in a single clutch. Sorted embryos that were not bisected were allowed to develop to score secondary axis induction.

### Total RNA isolation from gastrula stage embryos

Ten half-embryos were digested for 1 hr at 42°C in 1.2 mL of lysis buffer (0.5% SDS, 5 mM EDTA, 50 mM Tris pH 7.5, 50 mM NaCl) with 0.2 mg/mL proteinase K. Each sample was then extracted with an equal volume of acid phenol, and then phenol:choloform:isoamyl alcohol (25:24:1) using Eppendorf phase lock gel (PLG) tubes. Supernatants were precipitated with 120μl 3 M sodium acetate (pH 5.2) and 3 mL ethanol at -20°C for 1 hr. Pellets were spun down at 20800 g for 15 min, washed with 70% ethanol, and resuspended in 300μl RNase-free water. Solutions were then precipitated again by adding 60μl of 7.5 M lithium chloride, 50 mM EDTA, and incubating at -20°C overnight. The next day pellets were spun down, washed, and resuspended in 90μl RNase-free water. Solutions were DNased for 20 min at 37°C in 60μl of 1X DNase buffer, 2.5 mM DTT with 3μl RNase inhibitor and 2.4μl DNase (4.8U). Mixtures were then extracted with phenol:choloroform:isoamyl alcohol and PLG tubes, and supernatants were precipitated with 15μl 3 M sodium acetate and 375μl ethanol at -20°C for 1 hr. After spinning down and washing, pellets were resuspended in 11μl RNase-free water. 1μl was gel analyzed; 1μl was analyzed by a spectrophotometer. For each batch of ten half-embryos this procedure produced 10–30μg of total RNA.

### Microarray hybridization

RNA was biotin labeled and hybridized to Affymetrix GeneChip^® ^*Xenopus laevis *Genome Arrays according to the manufacturer's protocols (Affymetrix, Santa Clara, CA). The arrays contain 15,611 probe sets which represent approximately 14,400 transcripts.

### Microarray data analysis

Raw probe intensities (.cel files) from each oligonucleotide array were processed by the RMA algorithm [51], implemented by RMAExpress [52]. This method performs background correction and quantile normalization, followed by calculation of a PM-only log_2 _expression measure for each set of 16 probes. Raw .cel files as well as the processed log_2 _expression data can be freely downloaded from the NCBI GEO database as series GSE3368 [53]. Control sequences were filtered out leaving 15,491 probe sets. Log_2_-expression values from these genes were then tested for significant alteration between conditions by the rank products (RP) method, implemented by a perl script distributed by the method's author [21]. Subsets of genes selected by RP test criteria were subjected to hierarchical clustering by dChip [54,55], using the following options: replicates were averaged before clustering; all genes were standardized to each other, and stages were standardized separately; cluster calculation used Pearson correlation and centroid linkage.

### *Xenopus laevis *GO annotation

The Gene Ontology (GO) terms were annotated using the strategy proposed by Vinayagam *et al*. [48]. The method extracts possible GO terms for uncharacterized sequences by running BLAST against GO-mapped protein databases. Subsequently, suitable GO terms were predicted using a combination of multiple Support Vector Machines as well as a voting scheme devised for the purpose. Each prediction is associated with a confidence value to assess its reliability. Previously, this method was optimized only for molecular function GO terms.

We extended this approach to predict biological process terms and validated the prediction quality with 13 model organisms. Our data shows that biological process terms correlate less tightly with protein sequence similarity than molecular function. This is reflected in our dataset with more negative samples (terms inappropriate to the sequence) than positive ones (appropriate terms). Furthermore, the validation result shows a relatively poor correlation of the precision and accuracy values against the number of votes. However, at higher thresholds (more number of votes), a significant number of biological process GO terms were predicted with good precision (Additional file 5). Thus, considering only annotations with higher confidence values helps us to avoid misleading terms.

We applied this new biological process prediction approach to annotate *Xenopus laevis *contig sequences produced by The Institute of Genome Research (TIGR) *Xenopus laevis *Gene Index (XGI) [56], using the 39,558 contig sequences (excluding singletons) corresponding to XGI Release 8.0 (May 12, 2004). Our annotation system predicted GO terms for 15,649 of these sequences. After selecting only those terms predicted with confidence values of 70% or above, in order to remove uncertain predictions, 10,151 contigs remain with at least one biological process GO term. The annotations were then mapped to Affymetrix probe sets using TIGR's Resourcerer database [57]. At this point, some of the Affymetrix probe sets still held enormous numbers of GO terms. To ensure that we were only using the best available information for each gene, we restricted our annotation to ten terms for each probe set, selecting only the ten with the highest confidence values in cases where this limit was exceeded (Additional file 4). The EASE program was used to search gene clusters for enriched terms. Duplicate genes were removed prior to analysis. P-values were calculated using the Bonferroni corrected EASE score, which conservatively corrects for multiple testing [22].

### Whole mount *in situ *hybridization

Antisense probes were generated for *otx2 *and *foxA4a *(*pintallavis*) from pXOT30.1 [58] and Pintallavis/64T [59] plasmids, respectively. For the remaining genes chosen from the microarray clusters, publicly available clones were selected from the Unigene clusters used to build the probe sets, and then ordered from either NIBB [60] or IMAGE via Open Biosystems [61]. The following clones were used: Xl.3374.1.A1_at, XL456p16ex; Xl.13826.1.A1_at, XL051f16; Xl.13537.1.A1_at, XL027n24; Xl.15738.1.S1_at, XL159b23; Xl.19933.1.S1_at, IMAGE:4969205; Xl.12235.1.A1_at, XL218o09; Xl.610.1.S1_at, XL146e16; Xl.13019.1.S1_at, XL146o05; Xl.16672.1.S1_at, XL057a15; Xl.2466.1.A1_at, XL063j24; Xl.34.1.S1_at, XL064h24; Xl.620.1.S1_s_at, IMAGE:4404876; Xl.22607.1.S1_at, XL031h14; Xl.14812.1.S1_at, IMAGE:4959067; Xl.23634.1.S1_s_at, XL512p03ex; Xl.3435.1.A1_at, XL051c23; Xl.11129.1.A1_at, XL061l02; Xl.10684.1.A1_at, XL016j10; Xl.5940.1.A1_at, XL023i06; Xl.8933.1.A1_at, XL048m08; Xl.13925.1.A1_at, XL061i19; Xl.1643.2.S1_a_at, XL086e24; Xl.20670.1.S1_at, IMAGE:6643750; Xl.11148.1.A1_at, XL202e23; Xl.12869.1.A1_at, XL081f23; Xl.15362.1.A1_at, XL039i15; Xl.10415.1.A1_at, XL069c09; Xl.3529.1.A1_at, XL197l22; Xl.15745.1.A1_at, XL094f20; Xl.15270.1.A1_at, XL040d20; Xl.2208.1.A1_at, XL056a18; Xl.22609.1.S1_at, XL102p06; Xl.24155.1.A1_at, XL101m04; Xl.16395.1.S1_at, XL142j09; Xl.16206.1.A1_at, XL013l05; Xl.209.1.S1_at, XL060d01.

Whole mount *in situ *hybridization was performed as previously described [62]. In addition to the antisense *in situ *analyses, sense probes were also generated for the following clones, and used to conduct negative control *in situ *analyses: XL456p16ex, XL051f16, IMAGE:4969205, XL146o05, XL057a15, IMAGE:4404876, XL512p03ex, XL023i06, XL048m08, IMAGE:6643750, XL202e23, XL039i15, XL069c09, XL197l22, XL094f20, XL040d20, XL056a18, XL102p06, XL101m04, XL142j09, XL013l05, XL060d01. In all cases, the antisense *in situ *patterns were not present in the sense controls. In fact, all of the sense probes produced a similar pattern of background staining that varied only in intensity, which was characterized by faint general animal staining during blastula and gastrula stages, followed by diffuse general neural and neural crest staining during neurula stages.

### Real-time RT-PCR

Two additional set of samples were collected exactly as described for the microarray experiments at stage 11.5. Real-time RT-PCR was performed using an iCycler™ machine and iScript™ one-step RT-PCR kit with SYBR^® ^green (Bio-Rad, Hercules, CA). *ODC *and *otx2 *primer sequences, and anneal, extension and acquisition temperatures were used as described in Heasman *et al*. 2000 [63]. PCR was performed with a 30s annealing, 12s extension, and 30s acquisition. For each measurement the **Dor **sample was loaded at 100%, 50%, and 10% dilutions and used to define a standard curve; each condition is reported as a proportion of the **Dor **expression. Two technical replicates were conducted for each measurement and averaged. RT- controls were run and were negative in each case. Within each clutch, *Otx2 *expression was standardized by the *ODC *expression.

## Authors' contributions

ALH and JCB conceived and designed the *Xenopus *experiments and their analysis, and wrote the paper. ALH performed all the *Xenopus *experiments and analyzed the data. AV and SS provided the biological process GO annotation, and wrote the methods describing their generation.

## Supplementary Material

Additional File 1 **Supplementary scatter plots**. (A-F) Shows scatter plot comparisons of the conditions not shown in Figure 2. Log_2_-expression values were averaged between replicates and then plotted against the mean log_2_-expression of the stage-matched **Ven **condition. (A) **Nog **vs **Ven**, stage 10. (B) **Dkk **vs **Ven**, stage 10. (C) **Nog **vs **Ven**, stage 11.5. (D) **Nog+Dkk **vs **Ven**, stage 11.5. (E) **Dkk **vs **Ven**, stage 11.5. (F) **Dor **vs **Ven**, stage 11.5. Probe sets measuring two known organizer genes, *otx2 *(green) and *gsc *(red), are labeled within the plots (*otx2 *probe sets: Xl.1268.1.S1_at, Xl.3004.1.A1_at, Xl.11672.1.A1_at, and XlAffx.1.11.S1_at; *gsc *probe sets: Xl.801.1.A1_at, Xl.801.1.S1_at, and Xl.801.1.S1_s_at). (G-I) Shows scatter comparisons of selected single arrays, further illustrating the relative amounts of clutch variation and experimental variation. (G) **Dkk **vs **Ven**, clutch 3 stage 11.5. (H) **Dor **vs **Ven**, clutch 3 stage 11.5. (I) **Ven **clutch 3 vs **Ven **clutch 4, stage 11.5. Note that the R-square value in (I) is less than (G) and (H), showing greater clutch variation than experimental variation. Click here for file

Additional File 2 **Genes regulated by ectopic organizer signaling**. This tab delimited table contains information about the all of the genes which passed at least one RP method test above the 10% FDR cutoff in the **Nog**, **Nog+Dkk**, or **Dkk **conditions, when compared to **Ven**, at either stage. Figure 3 shows the results of hierarchical clustering of this list; genes are listed in the same order as the cluster. Columns: List Number, Affymetrix Probe Set, Unigene ID, Gene Title, Gene Symbol. Names and symbols were assigned by the Affymetrix NetAffx database [64]. Click here for file

Additional File 3 **Genes that show similar regulation in the full organizer conditions**. This tab delimited table contains information about the genes selected for our second list. Each gene was required to show either up-regulation or down-regulation that attained a RP test score less than 0.0006 in both the **Nog+Dkk **and **Dor **conditions, when compared to **Ven**. Genes are listed in the same order as the hierarchical cluster shown in Figure 7. Columns: List Number, Affymetrix Probe Set, Unigene ID, Gene Title, Gene Symbol. Names and symbols were assigned by the Affymetrix NetAffx database [64]. Click here for file

Additional File 4 **GO Biological Process annotation of the *Xenopus laevis *genome**. This tab delimited table contains the machine-generated GO Biological Process annotation used in this paper. Columns: Affymetrix Probe Set, Confidence Value, Biological Process ID, Biological Process Term. Click here for file

Additional File 5 **Precision and accuracy of the GO Biological Process annotation**. The accuracy and precision of the annotation test data are plotted against the number of votes. See Vinayagam *et al*. [48] for a description of the method used to produce these measures. Click here for file
