# Supplementary figures and images for: Genomic analysis of Xenopus organizer function
Source: BMC Dev Biol. 2006 Jun 6;6:27. doi: 10.1186/1471-213X-6-27 (PMC1513553; doi:10.1186/1471-213X-6-27)

**Additional Figure 1. Supplementary Scatter Plots**

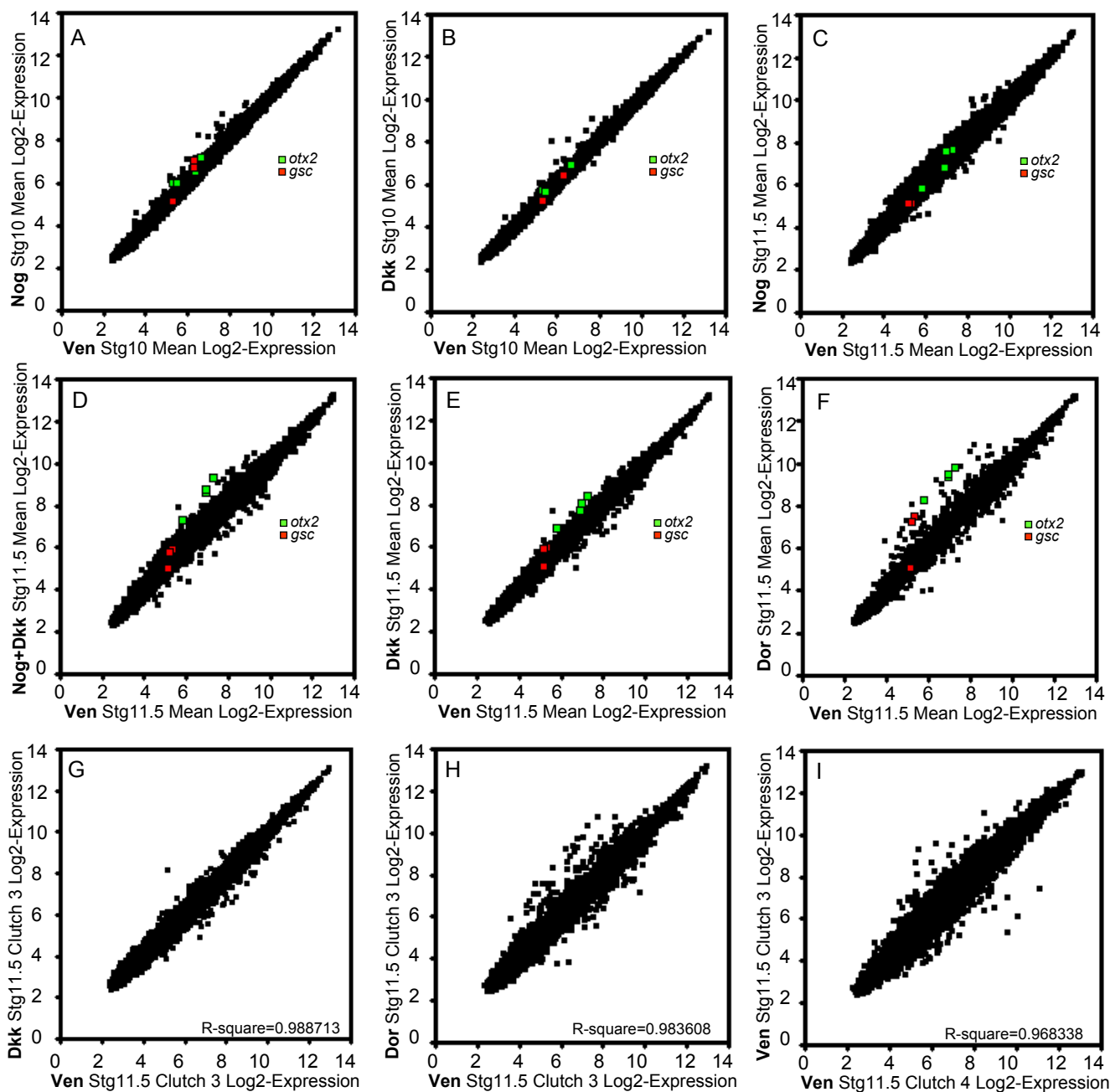

Supplement: Additional File 1 — Supplementary scatter plots. (A-F) Shows scatter plot comparisons of the conditions not shown in Figure 2. Log2-expression values were averaged between replicates and then plotted against the mean log2-expression of the stage-matched Ven condition. (A) Nog vs Ven, stage 10. (B) Dkk vs Ven, stage 10. (C) Nog vs Ven, stage 11.5. (D) Nog+Dkk vs Ven, stage 11.5. (E) Dkk vs Ven, stage 11.5. (F) Dor vs Ven, stage 11.5. Probe sets measuring two known organizer genes, otx2 (green) and gsc (red), are labeled within the plots (otx2 probe sets: Xl.1268.1.S1_at, Xl.3004.1.A1_at, Xl.11672.1.A1_at, and XlAffx.1.11.S1_at; gsc probe sets: Xl.801.1.A1_at, Xl.801.1.S1_at, and Xl.801.1.S1_s_at). (G-I) Shows scatter comparisons of selected single arrays, further illustrating the relative amounts of clutch variation and experimental variation. (G) Dkk vs Ven, clutch 3 stage 11.5. (H) Dor vs Ven, clutch 3 stage 11.5. (I) Ven clutch 3 vs Ven clutch 4, stage 11.5. Note that the R-square value in (I) is less than (G) and (H), showing greater clutch variation than experimental variation. [file 1471-213X-6-27-S1.PDF]

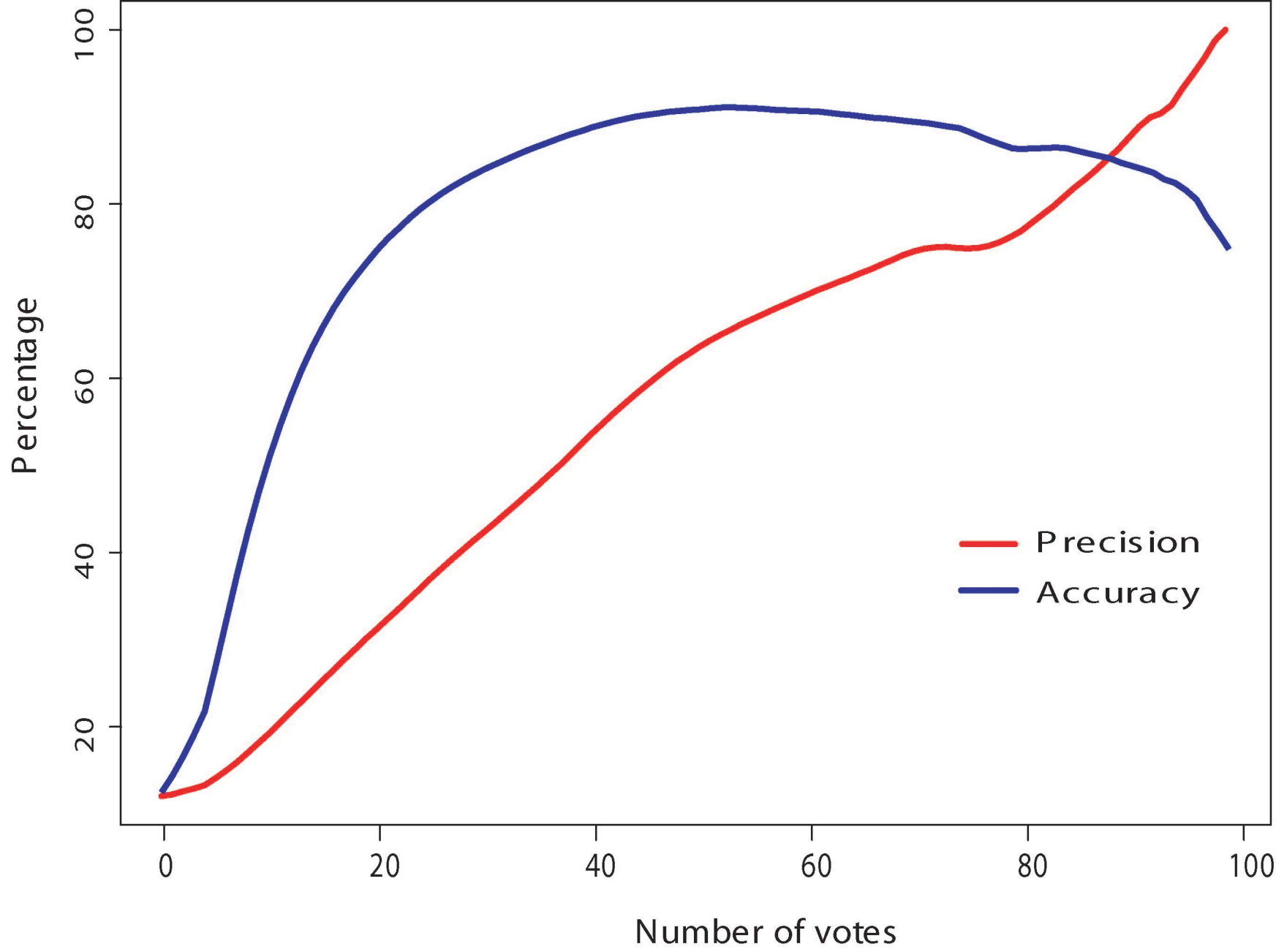

Supplement: Additional File 5 — Precision and accuracy of the GO Biological Process annotation. The accuracy and precision of the annotation test data are plotted against the number of votes. See Vinayagam et al. [48] for a description of the method used to produce these measures. [file 1471-213X-6-27-S5.pdf]
